# Pulmonary embolism presenting as non-ST elevation myocardial infarction: a case report

**DOI:** 10.4076/1757-1626-2-6328

**Published:** 2009-08-11

**Authors:** Amir Ahmad, Eduardas Subkovas, Jean Ryan

**Affiliations:** 1 Department of Cardiology, Glan Clwyd HospitalBodelwyddan Street, Rhyl, North Wales, LL18 5UJUK; 2 Library, Glan Clwyd HospitalBodelwyddan Street, Rhyl, North Wales, LL18 5UJUK

## Abstract

We describe a case which highlights the difficulties in diagnosing pulmonary embolism as it can mimic other conditions. In a patient with chest pain with raised troponin, a diagnosis of pulmonary embolism should also be considered as well if the clinical picture does not fit with myocardial infarction. Otherwise, the diagnosis of pulmonary embolism can be easily missed and patients may not receive appropriate treatment resulting in increased mortality.

## Case presentation

**Figure 1.**
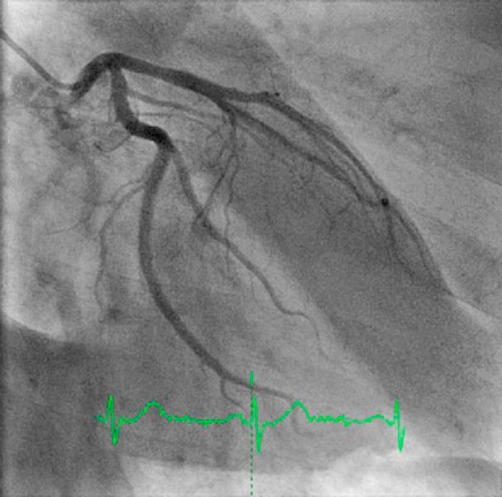
Angiogram showing normal coronary arteries.

A 55-year-old white British man very fit and well, presented with complaints of central chest tightness. It was non-radiating and lasted about 30 minutes. He usually walks two miles and does frequent cycling and swimming. He has not had chest pain previously and has always been in excellent health. He had a day case surgery for nasal polyp removal one week previously. He did not have any medical problems in the past and was not on any medications. He was a non-smoker. On examination all observations were normal and systematic examination was normal. All blood tests were normal. Electrocardiogram (ECG) and chest X-ray were normal. The Troponin-T test later came back raised at 0.14 micrograms per litre. A diagnosis of NSTEMI (non-ST elevated myocardial infarction) was made and he was started on appropriate treatment. The next day he had an in-patient coronary angiogram, which was normal (Figure 1). The cardiologist concluded that a diagnosis of myocardial infarction was unlikely in his case, all treatment was stopped and the patient was allowed to go home with no further follow-up.

**Figure 2.**
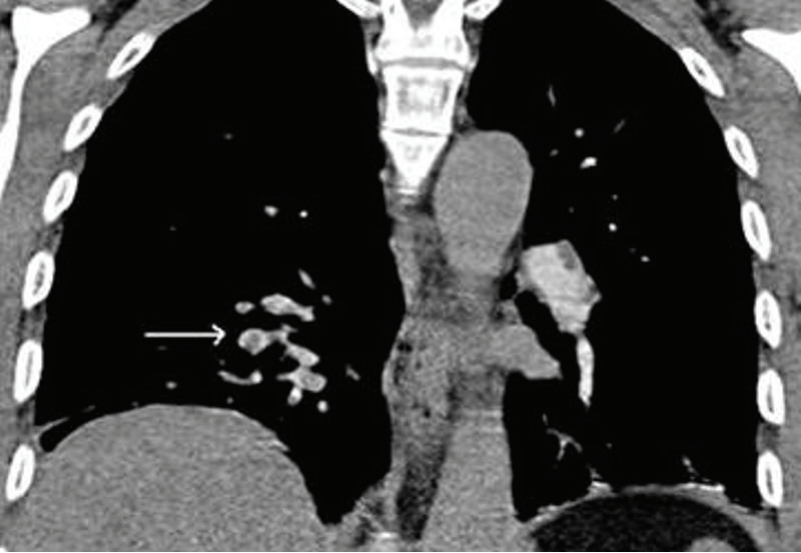
Arrow showing emboli in right pulmonary vasculature.

**Figure 3.**
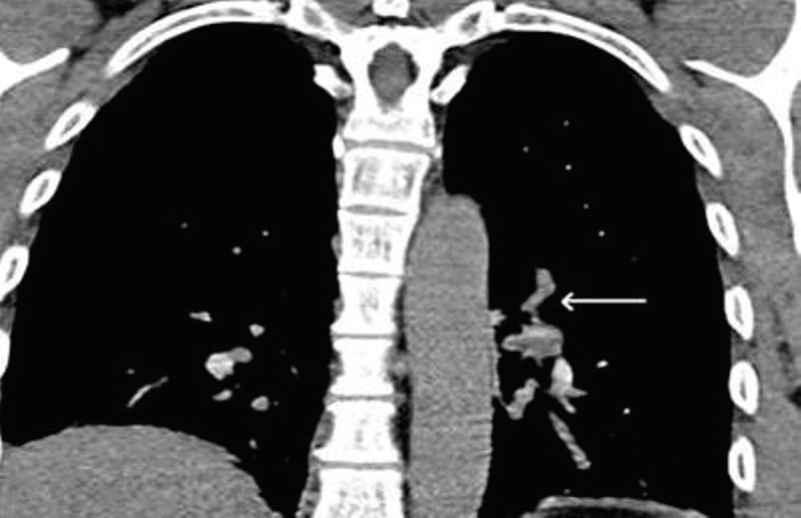
Arrow showing emboli in left pulmonary vasculature.

Four weeks later the patient again presented, this time with a history of left-sided chest pain. He described a sharp stabbing pain initially in the left rib cage and then in both sides later. Pain was made worse on inspiration, not related to posture or exertion and was only partially relieved with analgesics. He felt short of breath on walking which was unusual for a man of his fitness. On examination his observations were all normal. Systemic examination was normal. ECG and chest X-ray were normal. Arterial blood gases (ABG) showed- pH of 7.45, pCO_2_ of 4.3, PaO_2_ of 10.4, bicarbonate of 26 and saturation of 96%. Initially it was thought by the attending physician that the pain may be musculoskeletal. A diagnosis of pulmonary embolism (PE) was considered very unlikely in his case as he had no risk factors and was very fit, but in view of a slightly low PaO_2_ (though pCO_2_ was not low as would be expected in PE) for his level of fitness it was decided to do a CT pulmonary angiogram (CTPA) the next day. A troponin level on this occasion was normal but D-dimer came back as positive. No prediction score like Wells or Geneva was used at time of admission to determine the probability of PE. It is likely that even any such score had been used it would have shown a low probability given the absence of any risk factors for thromboembolism. To our surprise the CTPA showed bilateral pulmonary emboli in both the main pulmonary arteries and the segmental branches (Figures 2 and 3). The patient was started on warfarin following initial low molecular weight heparin. Once the INR was therapeutic, the patient was allowed to go home and was advised to continue on warfarin for 6 months.

## Discussion

The patient was initially diagnosed with myocardial infarction based on the nature of his chest pain and raised Troponin-T which is a very sensitive cardiac marker of injury. The diagnosis seemed so convincing that he had an urgent inpatient angiogram that was normal. The nature of the pain was different in the second admission, which was subsequently confirmed as due to the pulmonary emboli. It is likely that the Troponin rise in the first admission was due to the extensive PE which was discovered in the second admission. To suspect PE in his case was very difficult as he was remarkably fit for his age and had no apparent risk factors. Patients who have undergone significant operations (for example on the hip or abdomen) which predispose them to immobility are known to be at risk for PE [1]. Could a trivial day case nasal polyp operation (one week before the first admission) have been the predisposing risk factor in his case? Some coagulation disorders can increase the risk of thrombosis for e.g. factor Leyden mutation. The patient has to be off warfarin for at least three months before accurate thrombophilia testing can be done and our patient is waiting to undergo testing after his anticoagulation treatment is finished. This case highlights the difficulties in diagnosing PE as it can mimic other conditions. It underlines the need to keep a high index of suspicion even if a PE seems an unlikely possibility. Cardiac Troponins can be positive in both cases (acute myocardial infarct and pulmonary embolism), so one should be cautious in interpreting them if it does not fit with the overall clinical picture. Pruszczyk *et al.* in their study showed that 50% of their confirmed PE patients in the cohort had elevations in the Troponin level which was associated with an adverse prognosis [2]. To add to the diagnostic difficulty; sometimes both conditions (MI & PE) can show similar changes on the ECG in the form of T wave inversions. In such cases an echocardiogram can be useful as it can demonstrate a high RV:LV (ratio of end-diastolic dimension of the right and left ventricular chambers) which is a well recognized consequence of acute pulmonary arterial obstruction [3]. Treating our patient for myocardial infarction initially was probably the most prudent and appropriate thing to do given the history and elevated cardiac enzymes. But one important take-home message for doctors in training is that a patient with chest pain and an elevated cardiac Troponin should not be discharged home before eliminating the diagnosis of PE when an angiography has been found normal.

The other important learning point from this case for junior doctors is that when pulse oximetry appears deceptively normal in somebody complaining of shortness of breath it is advisable to perform a blood gas to confirm the presence of hypoxia and assess pulmonary shunting. Since respiratory alkalosis can left-shift the Haemoglobin-O_2_ dissociation curve, the SpO_2_ can wrongly be seen as normal. One should be aware that all the classical signs or risk factors might not be present in some patients with pulmonary embolism. Around 97% of patients with PE present complaining of at least one of the following: dyspnoea, tachypnoea or pleuritic chest pain. D-dimer test is useful in ruling out PE only in low probability cases and patients should undergo scan if they are deemed to be of high probability for having PE [4].

Therefore, think of PE in any patient presenting with chest pain or shortness of breath especially if the ECG and chest X-ray are normal and the patient is hypoxic [1,4].

**Figure 4.**
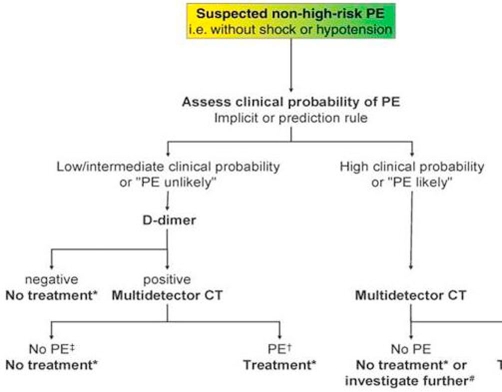
Proposed diagnostic algorithm for patients with suspected non-high-risk PE (i.e. without shock and hypotension) from the ESC guidelines.

Interpret any abnormal test results in the context of the full clinical presentation to avoid misdiagnosis. Risk prediction scoring (Table 1) though have some limitations if not used in the overall clinical context, should be used in all patients admitted with suspected PE to improve the accuracy of clinical judgement and investigations and stratify risk [5,6]. A diagnostic algorithm from the current European Society of Cardiology (ESC), guideline on the diagnosis and management of acute pulmonary embolism is shown in Figure 4 [7].

**Table 1.** Clinical prediction scores for PE: the revised Geneva score and the Wells score

Wells score		Revised Geneva score	
Variable	Points	Variable	Points
Predisposing factors		Predisposing factors	
Previous DVT or PE	+1.5	Age >65	+1
Recent surgery/immobilization	+1.5	Previous DVT or PE	+3
Cancer	+1	Surgery or fracture Within 1 month	+2
		Active malignancy	+2
Symptoms		Symptoms	
Haemoptysis	+1	Unilateral lower limb pain	+3
		Haemoptysis	+2
Clinical signs		Clinical signs	
Heart rate >100/min	+1.5	Heart rate	+5
		75-94/min	+3
		≥ 95/min	
Clinical signs of DVT	+3		
Clinical judgement			
Alternative diagnosis less than PE	+3	Pain on lower limb deep vein at palpation and unilateral oedema	+4
Clinical probability	Total	Clinical probability	Total
Low	0-1	Low	0-3
Intermediate	2-6	Intermediate	4-10
High	≥ 7	High	≥ 11
Clinical probability ( 2 levels)			
PE unlikely	0-4		
PE likely	0-4		

## Abbreviations

ABG: arterial blood gas
CTPA: computed tomography pulmonary angiogram
ECG: electrocardiogram
ESC: European Society of Cardiology
LV: Left ventricle
NSTEMI: Non-ST elevated myocardial infarction
PaO_2_: partial pressure of oxygen
pCO_2_: Partial pressure of carbon dioxide
PE: pulmonary embolism
RV: right ventricle
Spo2: oxygen saturation
